# Mycobiome temporal and functional dynamics in broilers: Ecological perspective on bacterial-fungal correlations and the effect of feed additives

**DOI:** 10.1016/j.psj.2025.106092

**Published:** 2025-11-10

**Authors:** Ana Fonseca, Sophia Kenney, John Boney, Erika Ganda

**Affiliations:** a Department of Animal Science, The Pennsylvania State University, University Park, PA, United States; b One Health Microbiome Center, The Pennsylvania State University, University Park, PA, United States

**Keywords:** Broiler, Age, Mycobiota, Feed additives, Functional perspectives

## Abstract

The gut mycobiome (the fungal component of the microbiome) of chickens, though less abundant than bacterial populations, plays a vital role in gut ecology, yet remains underexplored. This study investigated the temporal, dietary, and ecological factors shaping the broiler chicken excreta-associated fungal communities and their correlation with bacterial microbiota. A total of 320 Cobb 500 (1-day-old) chicks were raised for 21 days in 32 randomly allocated cages. Treatments consisted of four experimental diets: a Basal Diet, a Basal Diet with an Antibiotic (bacitracin methylene disalicylate), an Essential oils blend (oregano oil, rosemary, and red pepper), or a Probiotic (*Bacillus subtilis*). Shotgun metagenomic sequencing was performed on excreta samples collected at days 1, 10, and 21 to evaluate fungal diversity, composition, cross-kingdom correlation and functional profiling. The fungal community was dominated by Ascomycota and Basidiomycota across all treatments and time points. While alpha diversity metrics did not differ significantly between treatments (*P* > 0.05), fungal richness and evenness increased significantly over time (*P* < 0.05), indicating age-driven ecological succession. Beta diversity analysis revealed distinct age-related clustering patterns, with early dominance by *Candida albicans* and later shifts toward genera such as *Fusarium* and *Malassezia*. Feed additives exerted limited influence on fungal composition or diversity metrics, although clustering patterns suggested subtle treatment-specific effects over time. Cross-kingdom correlation analysis identified co-occurring temporal dynamics between the two microbial communities. *Candida* was positively correlated with *Streptococcus* and *Escherichia/Shigella* but negatively associated with beneficial genera like *Bifidobacterium* and *Faecalibacterium*. Additionally, microbial functional characteristics were observed in each treatment exhibiting metabolic features. Overall, this study demonstrates that excreta fungal succession in the broiler gut is primarily driven by host age and highlights the temporal plasticity of concurrent changes in fungal and bacteria communities. The findings underscore the importance of multi-kingdom ecological approaches to better understand gut health in poultry production.

## Introduction

The gastrointestinal (**GI**) microbiome of chickens is a complex, multi-kingdom ecosystem comprised of bacteria, fungi, viruses, and protozoa (Oakley et al., 2013; Shang et al., 2018).While bacterial communities have been extensively studied in different sample types for their role in nutrition, immunity, and growth, much less attention has been given to the fungal community - the mycobiome - in the gut (Davies et al., 2022). Recent advances in high-throughput sequencing of 18S or internal transcribed spacer (**ITS**) regions have enabled characterization of the chicken gut mycobiome, revealing relationships between the bacterial and fungal communities across GI tract segments (Burrows et al., 2025; Robinson et al., 2022; Shang et al., 2018).

The chicken gastrointestinal tract hosts a relatively limited yet stable fungal community, comprised of *Fusarium pseudonygamai, Candida albicans and Aspergillus flavus* (Robinson et al., 2022). This community is initially shaped by environmental exposures at hatch, including contact with egg incubators, eggshells, human handlers, and transportation containers. As chicks are introduced to the farm environment, their GI fungal composition evolves in response to external factors such as feed, litter, and air (Davies et al., 2022; Robinson et al., 2022). Common feed ingredients like corn and soybean meal often carry fungal genera such as Aspergillus, Penicillium, Fusarium, and Gibberella, which originate from agricultural fields and post-harvest storage conditions (Mansfield and Kuldau, 2007; Munkvold, 2003; Sugui et al., 2014). These feed-associated fungi may contribute to the early establishment and succession of broiler chickens’ gut mycobiota.

Probiotic feed additives, including *Bacillus, Lactobacillus*, and *Bifidobacterium*, along with phytogenic essential oils rich in bioactive compounds such as thymol and carvacrol, have emerged as promising alternatives to antibiotic growth promoters (**AGPs**) in poultry production. These additives have the capacity to modulate intestinal microbiota, including the mycobiota, with possible implications for gut health and overall performance (Adaszynska-Skwirzynska and Szczerbinska, 2019; Fonseca et al., 2024; Gumus and Gelen, 2023; Lee et al., 2017). Probiotics contribute to intestinal health by enhancing epithelial morphology, such as improving the villus-crypt ratio, and stimulating mucosal immune defenses, like elevated production of immunoglobulin A (**IgA**), lysozyme, and anti-inflammatory cytokines. Collectively, these may improve gut barrier integrity and inhibit colonization by pathogenic microbes through competitive exclusion (Bai et al., 2013; Khan et al., 2020; Rivera-Pérez et al., 2021; Tellez et al., 2020; Wu et al., 2011).

Essential oils are natural compounds extracted from plants with bioactive molecules that exert broad-spectrum antimicrobial effects (Simitzis, 2017). The antimicrobial activity, is largely attributed to their phenolic constituents which disrupt microbial membranes and inhibit the growth of opportunistic bacteria and fungi (Alagawany et al., 2018; Oniga et al., 2018; Salinas-Chavira and Barrios-García, 2024). Additionally, essential oils enhance antioxidant status and modulate inflammatory responses, thereby supporting gut homeostasis, immune function and performance (Ge et al., 2023; Oniga et al., 2018; Ruan et al., 2021).

Both probiotics and essential oils have been shown to reshape gut microbial community structure in poultry (Fonseca et al., 2024; Ge et al., 2023; Yang et al., 2020). Certain probiotic strains, such as Bacillus subtilis spores and Lactobacillus casei, may promote an anaerobic environment in the intestine, thereby stimulating the proliferation and competitive colonization of bacterial genera Lactobacillus, Faecalibacterium, and Bifidobacterium. These bacteria produce short-chain fatty acids (**SCFAs**), that can inhibit or reduce adhesion of pathogenic organisms such as Escherichia coli and Salmonella (Lee et al., 2015; Yang et al., 2012). In addition, probiotics may help control fungal overgrowth by degrading mycotoxins and reducing the residual presence and cytotoxicity through secretion of bacteriocins and organic acids (Chang et al., 2020; Liu et al., 2023). Active components in essential oils derived from oregano, lemongrass, thyme, and basil can modulate intestinal microbiota composition by increasing the relative abundances of bacterial families such as Actinobacteria, Bifidobacteriales, Enterococcaceae and Bacillaceae, that are helpful for overall microbial community maintenance (Corduk et al., 2013; Kim et al., 2023). Cinnamon, Palmarosa, Lemon eucalyptus and Honey myrtle has also been shown to have antifungal activity against opportunistic fungi, like *Candida albicans* in culture-based studies (Yuan et al., 2024).

Microbial interactions within the GI tract are driven by complex ecological dynamics, where microorganisms compete, cooperate, and coexist within ecological niches (Weiland-Bräuer, 2021). These interactions involve various ecological mechanisms, including changes in the physicochemical environment, the exchange and transformation of metabolites, cell signaling, chemotaxis, and gene transfer. Together, these mechanisms influence the selection of specific microbial genotypes which are products of co-evolutionary dynamics that promote adaptation and specialization, enabling microbes to occupy diverse ecological niches in the gut (Braga et al., 2016).

Each microbial taxon occupies a specific niche shaped by factors such as nutrient availability, oxygen levels, pH, and host-derived molecules (Pereira and David Berry, 2016). Competitive exclusion plays a critical role in maintaining microbial balance, as dominant taxa suppress the proliferation of others by outcompeting them for limited resources or producing antimicrobial compounds (Hibbing et al., 2010). At the same time, symbiotic relationships, including mutualism and commensalism, are essential for microbial stability and host health (Chow et al., 2010; Walter et al., 2018). Fungi-bacteria interactions can be intra- and/or interspecific and are mediated by cell-cell contact, and/or signaling molecules. For example, in vitro studies indicate that the contact between *Streptomyces* and *Aspergillus* reduce the production of aflatoxins by aspergilli. (Brakhage, 2013; Weiland-Bräuer, 2021).

Microbiome modulation has become an increasingly common strategy in poultry production, particularly through probiotic administration. Applying ecological principles can enhance our understanding of this microbial ecosystem, thereby improving the effectiveness of these interventions. Typically, probiotics are administered as either single strains or microbial consortia. Upon introduction into the GI tract, these inoculants must navigate various host-related barriers, including pH, diet, immune responses, and health status, to establish, proliferate, and yield a functional impact on the microbiome (Walter et al., 2018). Probiotic success is influenced by several factors, such as propagule pressure (i.e., dosage, delivery method, and frequency), microbial traits, adaptability, and phenotypic plasticity (Bajic and Sanchez, 2020). Moreover, the composition and functional capacity of the native microbial community plays a critical role in determining the successful persistence of probiotic strains (Albright et al., 2020, 2021). Notably, microbial communities with high species diversity and functional redundancy tend to be more resilient and competitive, thereby reducing the likelihood of successful colonization by newly introduced species (Weiland-Bräuer, 2021). Once established, the probiotic interacts with the resident community through different mechanisms, like competition, antagonism, mutualism, or predation, leading to either temporary or long-term microbiome modulation (Walter et al., 2018). However, because many interventions fail to account for these complex ecological interactions, microbiome modulation outcomes are often inconsistent and difficult to predict (Rocca et al., 2021).

Although recent studies have begun to describe the fungal composition of the chicken gut, significant gaps remain in our understanding of how the mycobiome develops and interacts with other microbial communities over time. Most research to date has focused on bacterial populations, possibly missing the ecological role of fungi and their relationship to host age and diet. This is a critical oversight, as emerging evidence suggests that the fungal mycobiome, though less abundant than bacteria, undergoes dynamic successional changes during the broiler production cycle. Understanding the balance between microbial correlations and their functional profiles is crucial for successfully manipulating gut communities through dietary interventions to improve animal health and productivity. Therefore, using shotgun sequencing, our objective in this study was to understand the temporal, dietary, and functional factors that shape the chicken excreta mycobiota and its correlations with the bacterial excreta microbiota.

## Materials and methods

### Experimental design

All experimental procedures were approved by the Institutional Animal Care and Use Committee (**IACUC**) of The Pennsylvania State University under protocol number PROTO202101779. The experiment was conducted at the Poultry Education and Research Center (**PERC**), and the performance and longitudinal excreta microbiota profile has been previously published in Fonseca et al. (2024). Here we focus on the evaluation of the excreta mycobiota through shotgun sequencing in three specific days including d1 (start of the experiment), d10 (change of diet from starter to grower), and d21 (the end of the experiment).

Briefly, one-day-old male Cobb 500 broiler chicks were used in a completely randomized block design. A total of 320 birds were individually weighed upon arrival and randomly allocated into 32 cages, with each cage housing 10 birds. The study included eight replicates per treatment, and all birds were reared under standardized management conditions from day 1 to day 21.

All diets were formulated based on corn and soybean meal in mash form, as previously described (Fonseca et al., 2024). The experimental treatments consisted of the following four dietary groups: (1) a commercial basal diet, (2) the basal diet supplemented with a *Bacillus subtilis* probiotic at 500,000 CFU/g (227 g/ton; Calsporin®, Calpis America, Inc., Peachtree City, GA), (3) the basal diet supplemented with an essential oil blend containing oregano oil, rosemary, and red pepper at 100 g/ton (Activo®, EW Nutrition, Adel, IA), and (4) the basal diet supplemented with bacitracin methylene disalicylate at 50 g/ton (BMD®, Zoetis, Parsippany, NJ), included as a conventional antibiotic growth promoter (AGP) control. Feed and water were provided *ad libitum* for the duration of the 21-day trial.

### Sampling collection

The chicken excreta (fecal or cecal droppings) were collected by placing sterile collection papers beneath each cage tray for a 90-minute period (starting at 7 am), the same time each day. Following collection, the papers were folded and transferred into sterile plastic bags (Whirl-Pak®, Avantor, Philadelphia, PA), then stored at –80 °C until further processing. Excreta samples from the boxes that birds were transported from the hatchery to PERC were collected as well and called Baseline.

### DNA extraction and sequencing

Prior to DNA extraction, all tubes and consumables used for sample storage and DNA extraction were certified DNA-free. The paper samples were randomized and thawed, followed by a standardized preprocessing procedure. Specifically, 50 mL of molecular-grade water (Intermountain Life Science, West Jordan, UT) was added to each Whirl-Pak® bag containing the collection papers with the chicken excreta. The contents were then homogenized for 2 min using a Stomacher® 400 Circulator. From the resulting homogenate, 4 mL was transferred into microcentrifuge tubes for storage. Genomic DNA was subsequently extracted using the KingFisher system in combination with the MagMAX™ Microbiome Ultra Nucleic Acid Isolation Kit (Thermo Fisher Scientific, Austin, TX, USA), following the manufacturer's protocol, with 200 µL of the homogenized sample used as input material. For each DNA extraction batch, a positive control comprised of ZymoBIOMICS Microbial Community DNA Standard (Zymo Research Corporation, Irvine, CA) and a negative control (reagents only) were included and carried through sequencing. As additional controls, one environmental (i.e., a paper placed inside of the cages without birds), and one autoclaved (a sample of the autoclaved paper batches) paper control were extracted and sequenced.

From the total of 320 birds sampled for genomic DNA extraction, a subset comprising baseline, days 1, 10, and 21 samples, along with negative and positive controls, was selected for shotgun metagenomic sequencing to evaluate the composition and temporal dynamics of the excreta-associated mycobiota over the three-time period selected. The quality and quantity of the genomic DNA were assessed with a spectrophotometer (Nanodrop, Thermo Fisher Scientific Inc., Waltham, MA, USA) then, shipped on dry ice to Novogene Corporation Inc (Sacramento, CA) for library preparation and whole-genome sequencing on the NovaSeq 6000 platform (Illumina, CA, USA), returning 2 × 150 paired-end reads.

### Statistical analysis and bioinformatics

Quality control and length trimming were performed using FastQC v0.11.9, and Trimmomatic v0.39 tools (Bolger et al., 2014; Chen et al., 2018). Via the AMRPlusPlus bioinformatic pipeline (Bonin et al., 2023), paired-end reads were trimmed to remove low-quality bases and adapter sequences. A sliding window approach was applied, whereby a 4-base window was trimmed if the average Phred quality score dropped below 15. Leading and trailing bases were removed if their Phred scores were below 3. Reads shorter than 36 bases after trimming were discarded. Adapter contamination was removed using the TruSeq3-PE-2.fa adapter file provided by Trimmomatic package (Chen et al., 2018). Host read decontamination was performed with default parameters in the same pipeline, using a *Gallus gallus* genome assembly (GCA_016699485) for reference.

Following host read removal, taxonomic classification was performed using Kraken2 v2.1.3 (Wood et al., 2019). For bacterial profiling, reads were aligned against the standard Kraken2 database which includes complete bacterial, archaeal, and fungal genomes from RefSeq (built March 2025). To improve the accuracy of taxonomic abundance estimates, Kraken2 output was processed with Bracken v2.8 (Lu et al., 2017), and BIOM tables were generated. Remaining unclassified reads were subsequently mapped to Kraken2′s comprehensive fungal library, which includes complete RefSeq fungal genomes and proteins (built April 2025). The resulting Kraken2 classification reports were converted into BIOM format as above. Both bacterial and fungal biom tables are available at https://github.com/aff30/Poultry-Mycobiome-. All downstream analyses were performed using R software version 4.4.2.

To assess temporal dynamics and feed additive effects on broiler excreta mycobiota, statistical comparisons were performed for alpha diversity metrics (within-sample evenness or richness), beta diversity (between-sample diversity or community structure), and differential relative abundance of fungal genera across age and treatments. Alpha diversity was calculated as the Shannon (considers evenness and richness) and Observed ASVs indexes (unique ASVs). Kruskal-Wallis test was used to compare alpha diversity across treatment groups, and age. Dunn’s test was performed for multiple comparisons and *p*-values were adjusted by using the BH method.

Fungal community counts were center log-ratio (CLR) transformed and visualized in a principal coordinates analysis (PCA) at the genus level using the microViz package (Barnett et al., 2021). Beta diversity differences were analyzed with permutational multivariate ANOVA (PERMANOVA), with age, and treatment using the Adonis test with 10,000 permutations on Aitchison distances. Pairwise comparisons were calculated using the vegan package (Oksanen et al., 2022). Differential relative abundance, which determines if the fractional abundance of a taxa that differs between a pairwise comparison, was performed between age and treatments using a Dirichlet-multinomial model to infer abundance from counts in the ALDEx2 R package (Nixon et al., 2022). Differences at *P* ≤ 0.05 were deemed to be significant.

To assess potential ecological correlations between fungal and bacterial communities, Spearman correlation analyses were performed on relative abundance data derived from bacterial and fungal datasets. Microbial count data were first transformed to relative abundances and aggregated at the genus level using the phyloseq R package (McMurdie and Holmes, 2013). The top 15 most abundant genera from both fungi and bacteria were retained based on mean relative abundance across all samples. For each treatment versus age group, we computed pairwise Spearman correlation coefficients between fungal and bacterial genera with the Hmisc R package. Correlations with a *P* ≤ 0.05 were considered statistically significant and the results were visualized using heatmaps. All data visualizations were performed with the microViz, ggplot2, pheatmap R packages, and annotated using Adobe Illustrator (Barnett et al., 2021; Wickham, 2009).

To evaluate the metabolic potential of the microbial communities, after Kraken2 classification the classified reads from both bacterial and fungal database were functionally annotated against the Kyoto Encyclopedia of Genes and Genomes **(KEGG)** database. Functional profiling was performed using FuncProfiler v.3.8.20 (Hera et., 2024)**,** which assigns KEGG Orthologs **(KOs)** to predicted coding sequences and maps them to corresponding KEGG pathways. This approach enabled the identification and quantification of genes associated with secondary metabolism**,** environmental adaptation, and other microbial functional categories. The resulting KO abundance tables were converted to BIOM format for downstream analyses. To assess differential relative abundance of KEGG Orthologs among dietary treatments, we used the ALDEx2 package in R. Statistical significance was determined using the Kruskal-Wallis test with Benjamini-Hochberg correction to control for multiple comparisons.

## Results

### Sequencing results

Before sequence read preprocessing, the 214 samples resulted in an average of 26,456,463.05 raw reads per sample. After quality control and host read removal, a total of approximately 22.8 million reads per sample were subjected to taxonomic classification. Of these, an average of 16,200,975.86 reads were successfully classified using a bacterial reference database through Kraken2. The remaining 6,631,691.37 reads, which were unclassified at this stage, were subsequently analyzed using a fungal-specific database encompassing a total of 91 taxa in the final dataset to characterize the mycobiome. The raw sequences are available at PRJNA928060.

### Fungal composition classification

**Fig. 1:**
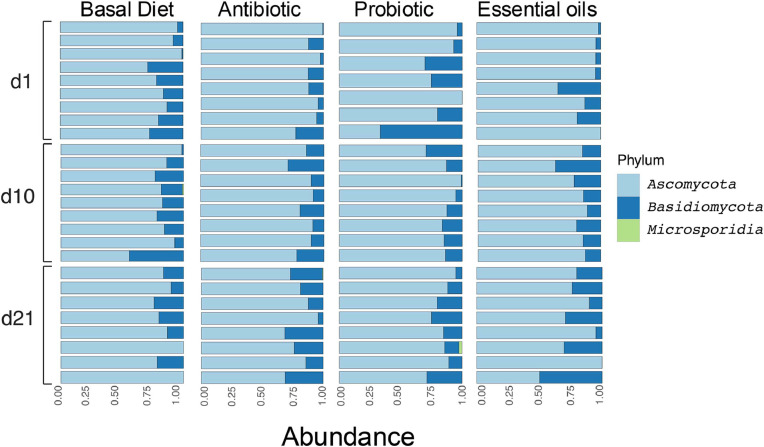
Relative abundance (%) of the most abundant fungal taxa among treatment groups (Basal Diet, Probiotic, Essential Oils, and Antibiotic) by age at the phylum level. Each color represents one phylum group, numbers in the x axis indicate relative abundance and y axis day of study. Each horizontal bar represents one sample.

Across all treatment groups, Ascomycota (84.02 %; ± 11.50 %), and Basidiomycota (15.93 %; ± 11.50 %), were the most prevalent phyla followed by Microsporidia (0.039 %; ± 0.25 %) (Fig. 1). When considering relative abundance of fungal taxa by age (independent of diet), we observed a slight decrease of Ascomycota from d1(86.06 %; ± 13.88 %) to d21 (82.28 %; ± 11.13 %) and an increase of Basidiomycota, d1(13.94 %; ± 13.88 %) and d21 (17.63 %; ± 11.15 %). Broilers fed Antibiotic had the largest relative abundance of Ascomycota (85.00 %; ± 9.13 %) followed by Basal Diet (84.35 %; ± 10.24 %), Essential oils (83.54 %; ± 12.86 %), and Probiotic (83.13 %; ± 14.00 %) diet groups. Conversely, Basidiomycota was more abundant in the Probiotic group (16.75 %; ± 14.03 %) than in other groups: Essential oils (16.45 %; ± 12.86 %), Basal Diet (15.61 %; ± 10.23 %), and Antibiotic (14.97 %; ± 9.10 %). Microsporidia were present at low abundances in most groups (Probiotic: 0.10 %; ± 0.48 %; Basal Diet: 0.036 %; ± 0.18 %; Antibiotic: 0.021 %; ± 0.10 %), except the Essential oils group wherein it was not detected.

At family level, the most relatively abundant families found across all data were *Debaryomycetaceae* (16.20 %; ± 26.80 %), *Nectriaceae* (14.50 %; ± 12.49 %), *Aspergillaceae* (12.65 %; ± 11.20 %), *Malasseziaceae* (8.62 %; ± 9.38 %) and *Saccharomycetaceae* (7.01 %; ± 7.32 %). We observed changes in these relative abundances over time. At d1 *Debaryomycetaceae* (38.17 %; ± 32.80 %) was the most relatively abundant family, followed by *Nectriaceae* (19.61 %; ± 17.15 %), and *Aspergillaceae* (14.45 %; ± 14.40 %). By d10, *Aspergillaceae* (12.80 %; ± 8.37 %) was most relatively abundant, followed by *Saccharomycetaceae* (9.92 %; ± 5.85 %) and *Nectriaceae* (9.92 %; ± 8.28 %). Later, at d21, *Nectriaceae* (14.11 %; ± 8.30 %) and *Malasseziaceae* (12.58 %; ± 10.46 %) were the most relatively abundant families followed by *Aspergillaceae* (10.66 %; ± 10.07 %). When considering treatment, *Debaryomycetaceae* (20.02 %; ± 30.19 %) was most relatively abundant in the Basal Diet, followed by Essential oils and Antibiotic that had similar relative abundances (17.15 %; ± 21.91 %), and Probiotic (14.43 %; ± 23.12 %).

**Fig. 2:**
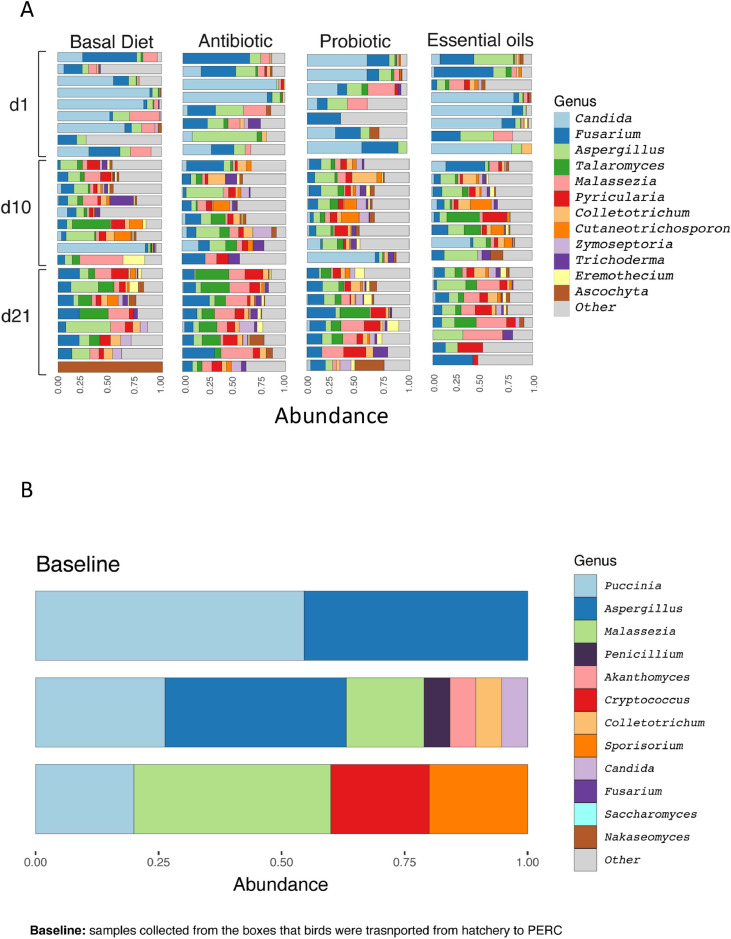
(A) Relative abundance (%) in three different ages (1, 10, and 21 days) at the genus level. Each color represents one genus, numbers in the x axis represent the relative abundance and y axis represent day of study. Each horizontal bar represents one sample. (B) Relative abundance (%) of the Baseline (samples collected from the boxes that birds were transported from hatchery to PERC at the genus level. Each color represents one genus, numbers in the x axis represent the relative abundance and y axis represent each box. Each horizontal bar represents one sample. The 12 most abundant genera were used, and the remainder were included as “Other”.

The composition and distribution of fungal genera display some variability across treatments (Fig. 2A). *Candida* (15.50 %; ± 26.90 %), *Fusarium* (14.50 %; ± 12.49 %), and *Aspergillus* (11.90 %; ± 10.46 %) were the most abundant genera across all age and treatments. Specifically, at d1, *Candida* (37.50 %; ± 33.04 %), *Fusarium* (19.61 %; ± 17.15 %), and *Aspergillus* (13.15 %; ± 12.66 %) were the most relatively abundant genera. However, at d10 and d21 respectively, *Aspergillus* (12.53 %; ± 8.46 %) and *Fusarium* (14.11 %; ± 8.30 %) become the most relatively abundant. Regarding diet, Probiotic and Basal Diet groups had higher relative abundances of *Candida* (Probiotic: 17.08 %; ± 29.91 %; Basal Diet: 15.41 %; ± 27.38 %). *Fusarium* was relatively most abundant in Antibiotic (16.39 %; ± 14.45 %) and Probiotic (14.37 %; ± 9.07 %) fed birds. *Aspergillus* was most abundant for Essential oils (14.63 %; ± 11.11 %) fed birds followed by Antibiotic (11.96 %; ± 14.29 %), Basal Diet (11.31 %; ± 9.93 %), and Probiotic (9.79 %; ± 5.80 %) fed birds. Other genera such as *Malassezia, Talaromyces, Penicillium* and *Saccharomyces* were more evenly distributed among all the treatment groups but changed the dynamic overtime (Fig. 2A). The presence of rare genera such as *Eremothecium, Ascochyta*, and *Zymoseptoria* was more pronounced in the Basal Diet and Probiotic groups. In the Baseline samples (box samples), *Aspergillus* (21.05 % ± 18.97 %) was the most relative abundant genus followed by *Puccinia* (21.05 % ± 13.92 %), *Malassezia* (8.77 % ± 8.03 %), *Penicillium* (1.75 % ± 3.03 %) and *Candida* (1.75 % ± 3.03 %) as shown in Fig. 2C.

At d1 the most abundant species were *Candida albicans* (37.34 %; ± 33.16 %), *Malassezia restricta* (7.19 % ± 8.64 %) and *Fusarium graminearum* (5.14 %; ± 7.88 %). We observed a decrease in *Candida albicans* from 8.46 %; ± 18.98 % at d10 to 0.25 %; ± 0.79 % by d21. Across all treatments, the most predominant species was *Candida albicans*, with higher proportions in the Basal Diet (19.30 %; ± 30.52 %), followed by Essential oils (16.30 %; ± 29.44 %), and Probiotic (14.43 %; ± 23.12 %), and Antibiotic groups (10.49 %; ± 24.71 %). Interestingly, the second most abundant fungal species vary between treatment groups wherein *Malassezia restricta* and *Malassezia vespertilionis* were most abundant in the Basal Diet (5.77 %; ± 10.36 %), and Essential oils (5.40 %; ± 11.01 %) while *Aspergillus rugulosus* was relatively more abundant for Antibiotic (6.99 %; ± 8.81 %) and Probiotic (4.30 %; ± 5.65 %) groups. Complete tables with the relative abundances of taxa in each sample at the phylum, family, genus, and species levels are provided on GitHub (https://github.com/aff30/Poultry-Mycobiome-).

### Fungal alpha diversity analysis

**Fig. 3:**
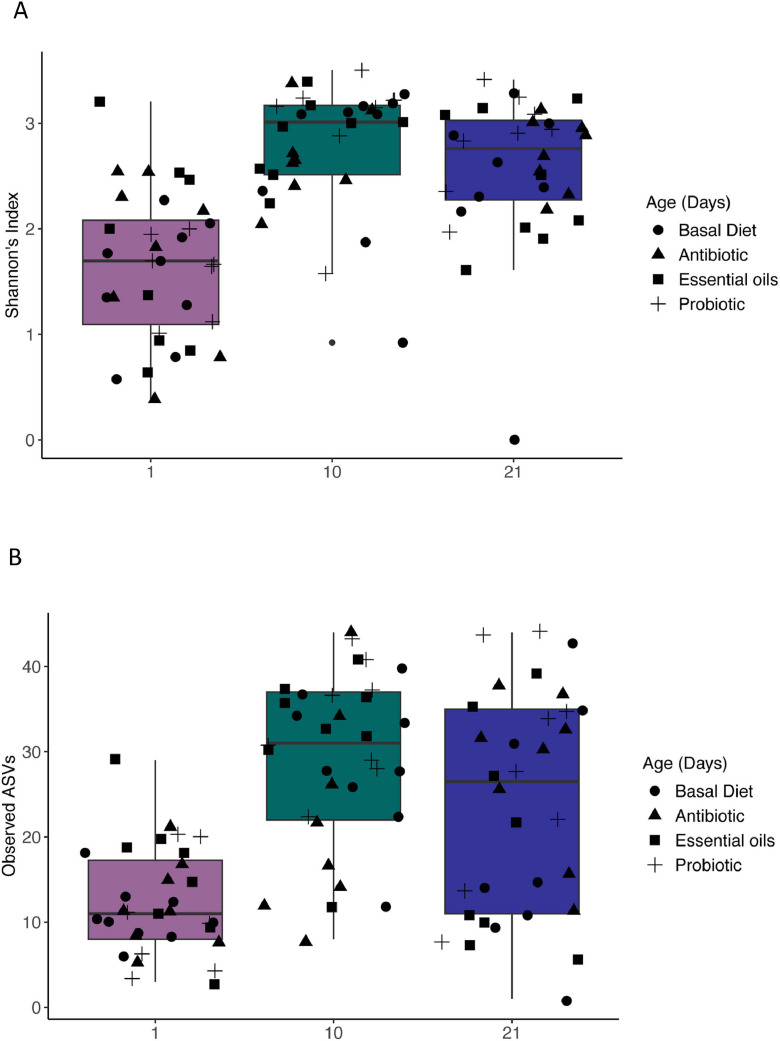

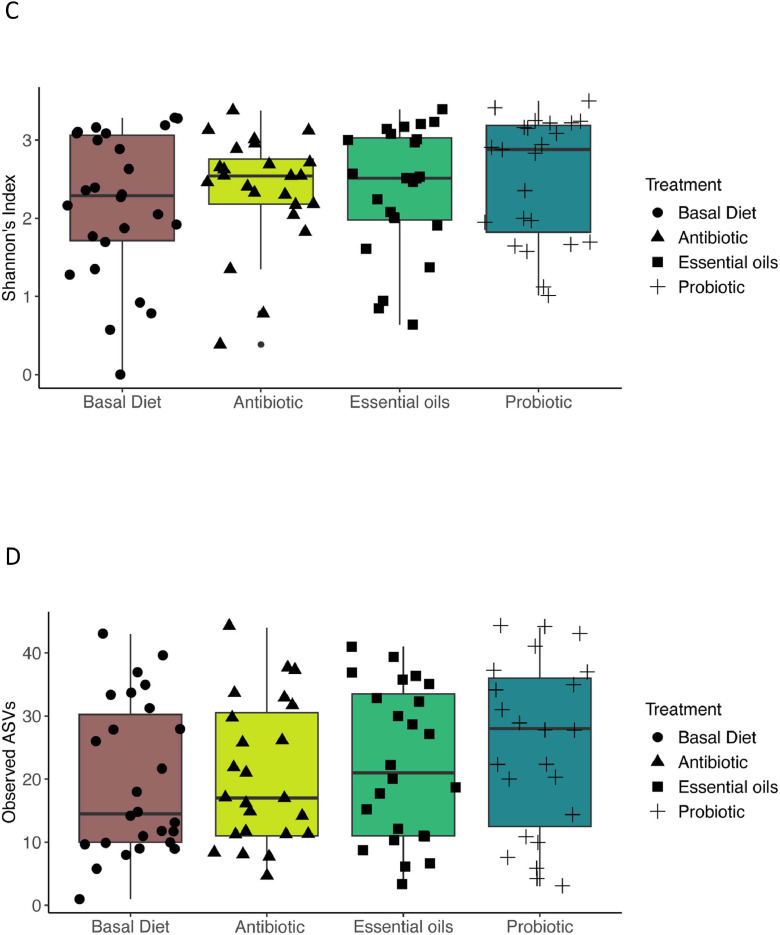
Alpha diversity comparisons. Alpha diversity calculated as the Shannon and Observed ASVs indexes for age and treatment groups were compared with Kruskal−Walli’s test. Boxplots of alpha diversity (Shannon index – A and Observed ASVs − B) of days 1, 10, and 21. Colors represents the age in days and black shapes represent the treatments. Boxplots of alpha diversity (Shannon index – C and Observed ASVs − D) of treatment groups (Basal Diet, Antibiotic, Essential oils, Probiotic). Colors and points represent treatments. Each box plot indicates the interquartile range (IQR) with the median represented by the horizontal line within the box, while whiskers extend to display the full range of the data excluding outliers, which are depicted as individual points.

Alpha diversity metrics, including Shannon, and observed ASVs, were calculated to assess differences across age and treatment groups. We saw significant variation in alpha diversity within bird age (Fig. 3A). Specifically, diversity increased with age with later time points (10 and 21) showing higher richness and evenness (Shannon index, *P* < 0.05; Observed ASVs, *P* < 0.05). These findings suggest temporal dynamics consistent with fungal succession in the excreta mycobiome. When considering feed additives (Fig. 3C and D), no significant differences in alpha diversity were observed between treatments (*P* > 0.05).

### Fungal beta diversity analysis

**Fig. 4:**
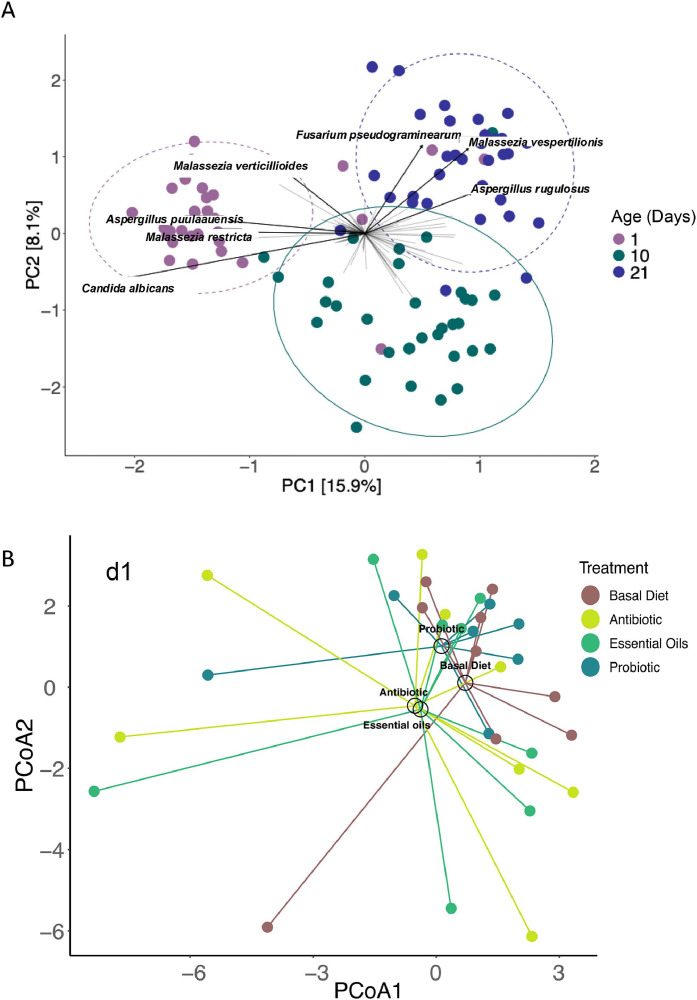

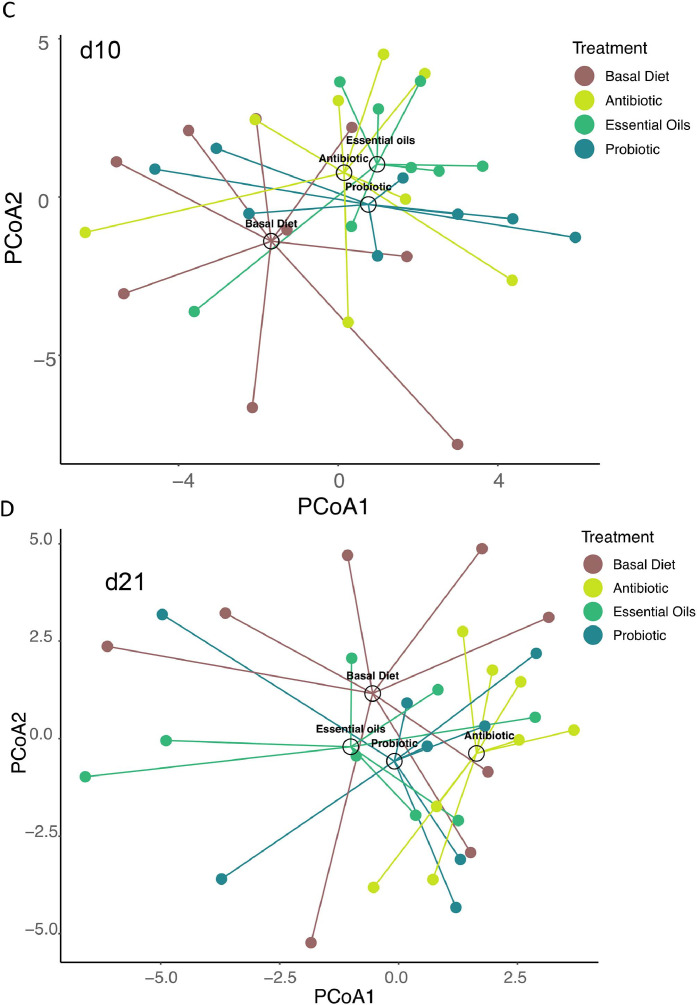
Beta diversity comparisons. Beta diversity differences were analyzed with permutational multivariate ANOVA (PERMANOVA) with time, and treatment using the Adonis test with 10,000 permutations on Aitchison distances. (A) Principal component analysis (PCA) of centered log-ratio-transformed microbiome data at genus level showing the 7 top fungal loadings. Samples are representing three different time points of the experiment d1, d10 and d21 shown by the color. (B-D) Principal coordinates analysis (PCoA) centered log-ratio-transformed microbiome data at the genus level based on centroids dissimilates. Treatments are represented by color; each centroid is shown by treatment name and age days are represented by panel (B = d1; C = d10 and D = d21).

Clear clustering patterns emerged across age groups (d1, d10, and d21), indicating that the fungal community composition shifts over time (*P* < 0.01) (Fig. 4A). Samples collected at d1 formed a distinct cluster, while those from d10 and d21 showed partial overlap. Species-level loadings identified the taxa driving this variation. *Candida albicans* and *Malassezia restricta* were predominantly associated with early-age samples, whereas *Aspergillus rugulosus* and *Fusarium pseudograminearum* contributed more to variation at later time points.

To further assess the impact of dietary treatments over time, principal coordinates analysis (PCoA) graphs based on centroids dissimilates were constructed (Fig. 4B-D). Distinct clustering patterns were evident across treatments by age, demonstrating that diet supplementation and age influenced the overall structure of fungal communities. Although no significant differences were seen in centroid location according to treatment at d1 (*P* = 0.75), the dispersion is greater in chickens fed Antibiotic. Visually, Antibiotic and Essential oils groups overlapped in the ordination space, suggesting similar fungal compositions while Probiotic and Basal Diet groups clustered closely while remaining distinct from the former two (Fig. 4B). By d10, (Fig. 4C) all feed additive (Probiotic, Antibiotic and Essential oils) groups had shifted away from the Basal Diet, but no differences were observed in centroid location at that day (*P* = 0.14). No differences were seen at d21 (*P* = 0.17, Fig. 4D); however, centroids representing each treatment were dispersed.

### Differential relative abundance

**Fig. 5:**
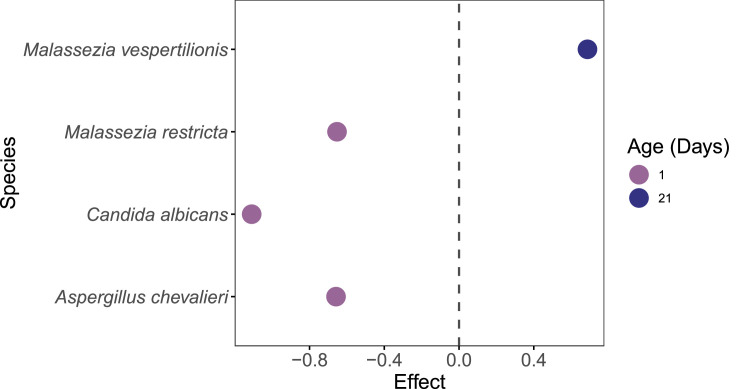
Differential relative abundance. Data were assessed by using ALDEx2 software, applying a t-test between day 1 (d1) and day 21 (d21) samples on CLR transformed data at the species level, while accounting for sample type variance. The effect size is shown for each fungal species with their respective name. Significant differences are shown in purple or blue circles (positive log2 fold change is more relatively abundant at age days and negative log2 fold change is less relatively abundant at age days comparing day 1 to day 21). Dashed vertical line at 0.0 marks the neutral effect.

Supplementing broiler diets with Probiotic, Antibiotic or Essential oils did not yield significant differences in the relative abundance of fungal species when compared to the Basal diet. Age-related effects were nonetheless observed, with specific differences between day 1 and day 21 (Fig. 5). *Malassezia vespertilionis* was significantly more abundant at d21 (effect size = 0.68, *P* < 0.05), indicating a positive relationship with bird development or change of diet and nutritional requirements. Conversely, *Malassezia restricta* (effect size = −0.65, *P* < 0.05), *Candida albicans* (effect size = −1.10, *P* < 0.05), and *Aspergillus chevalieri* (effect size = −0.65, *P* < 0.05), were relatively less abundant on day 1 compared to d21, pointing to early colonization dynamics. These findings highlight the impact of age on excreta-associated fungal mycobiota composition in broiler chickens, while conversely showing that feed supplementation strategies had minimal influence on fungal taxa.

### Correlation analysis between the bacterial and fungal microbiota

**Fig. 6:**
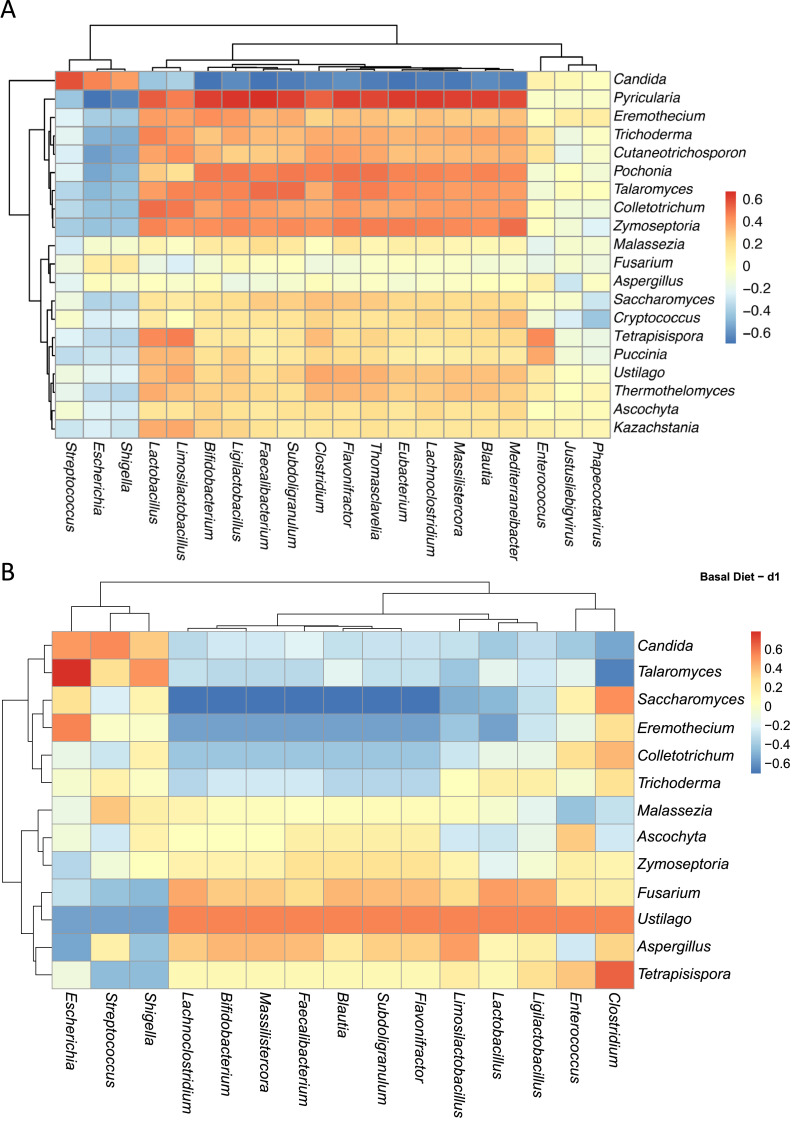

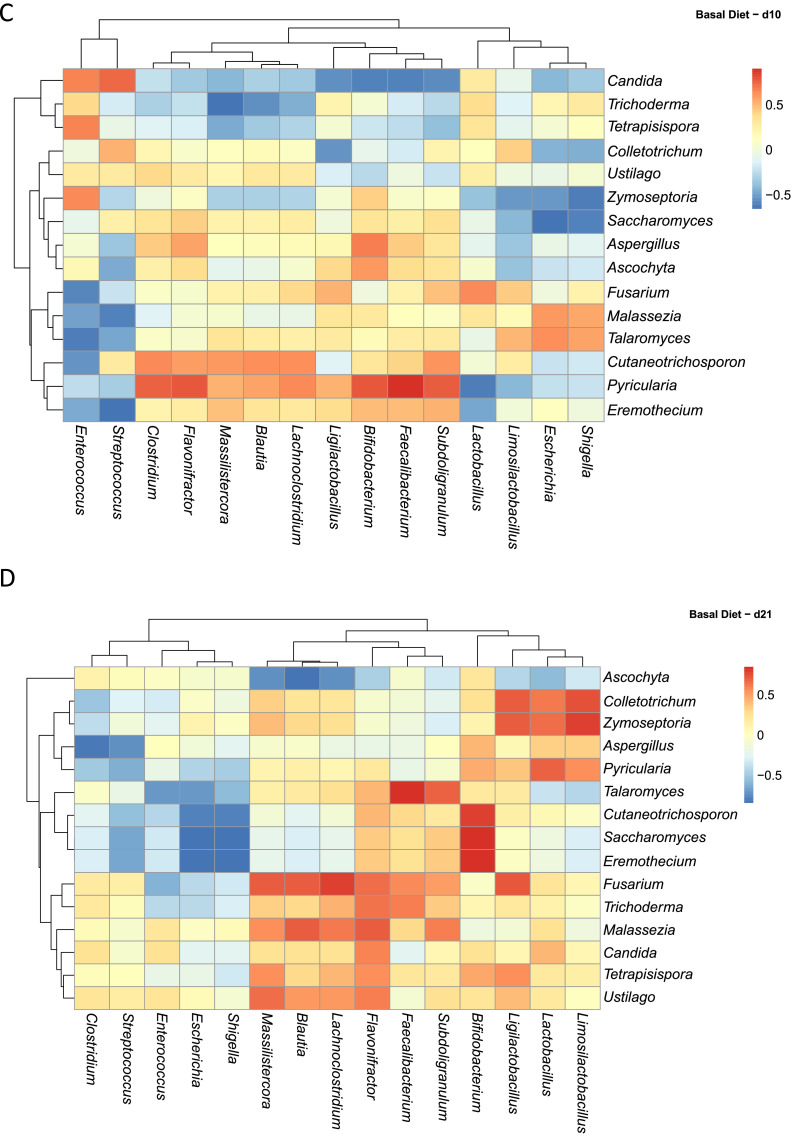

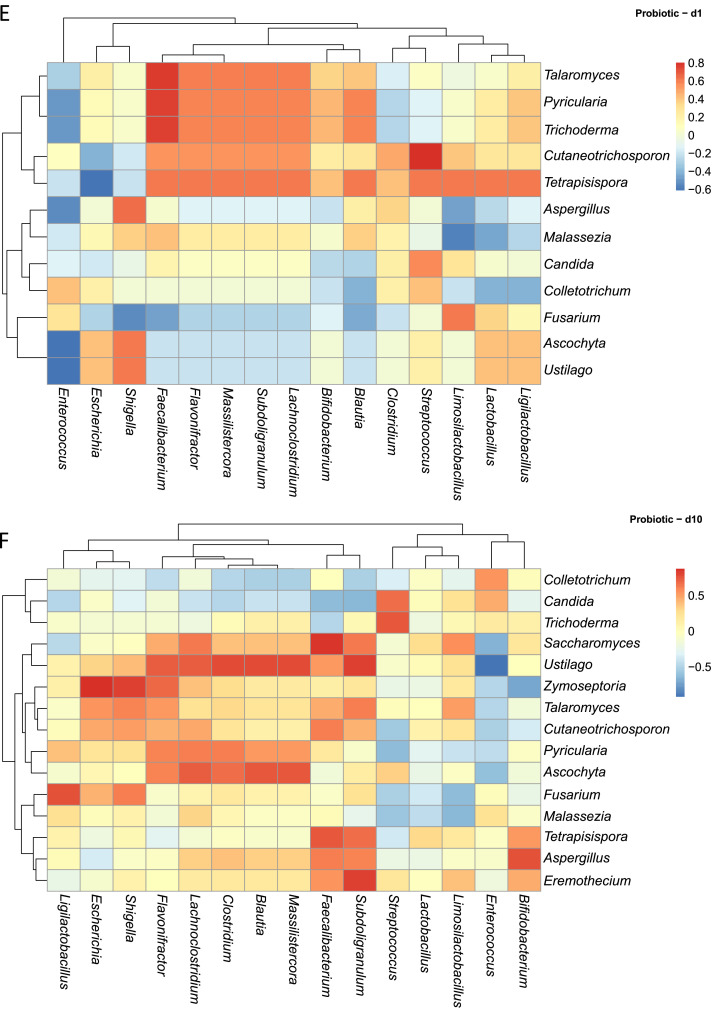

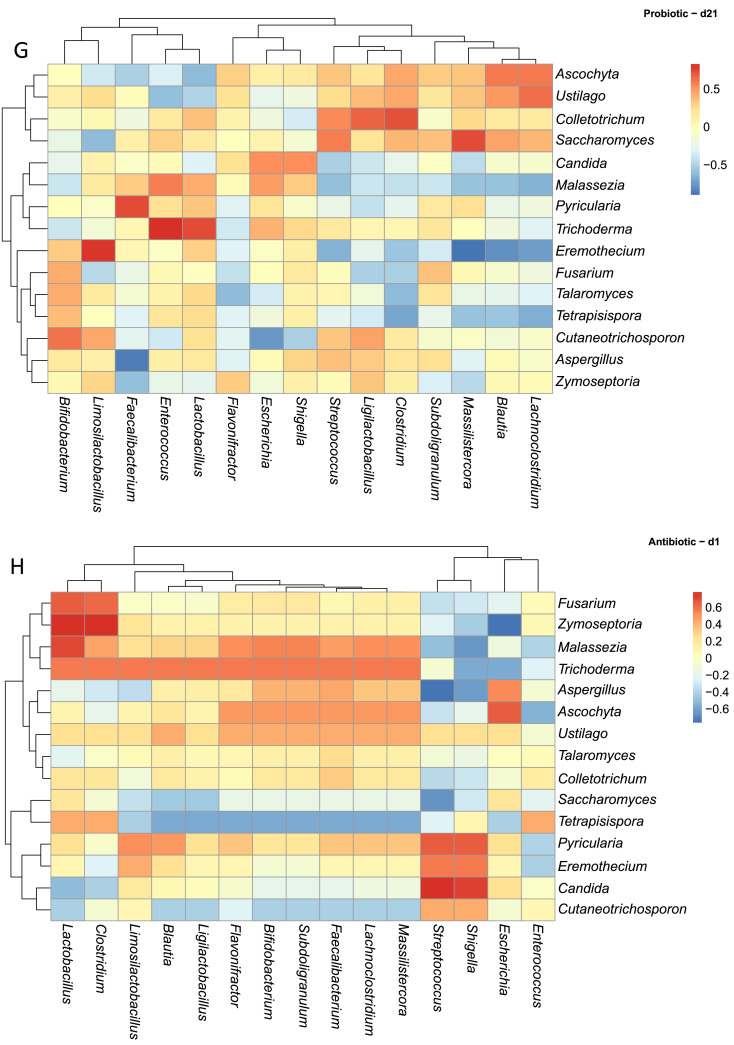

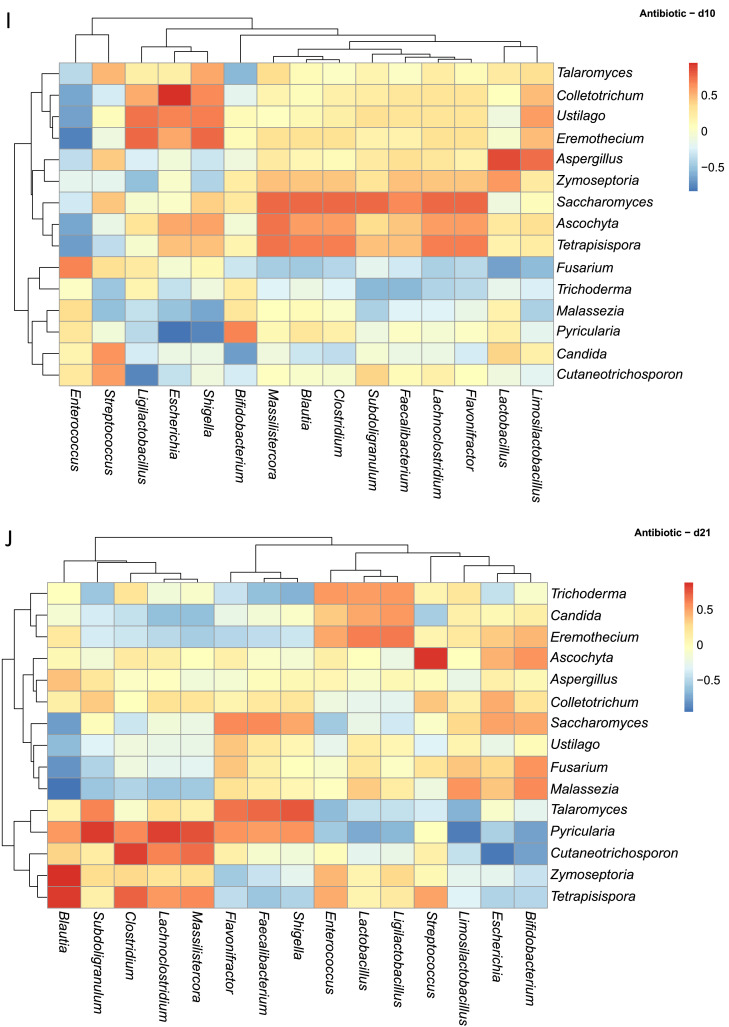

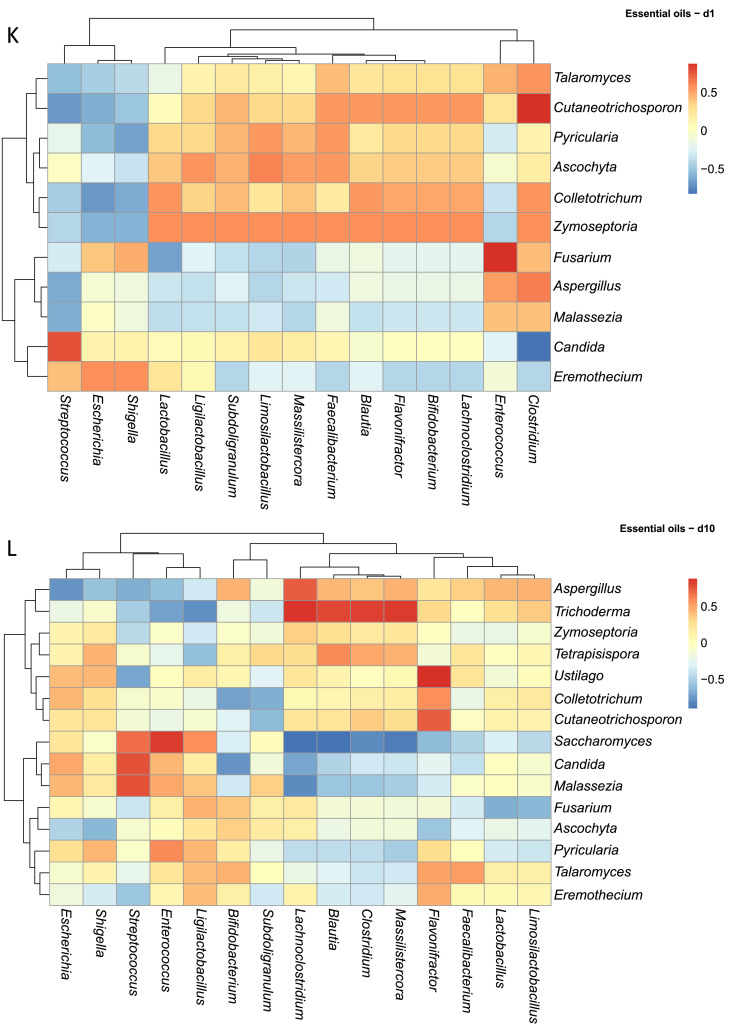

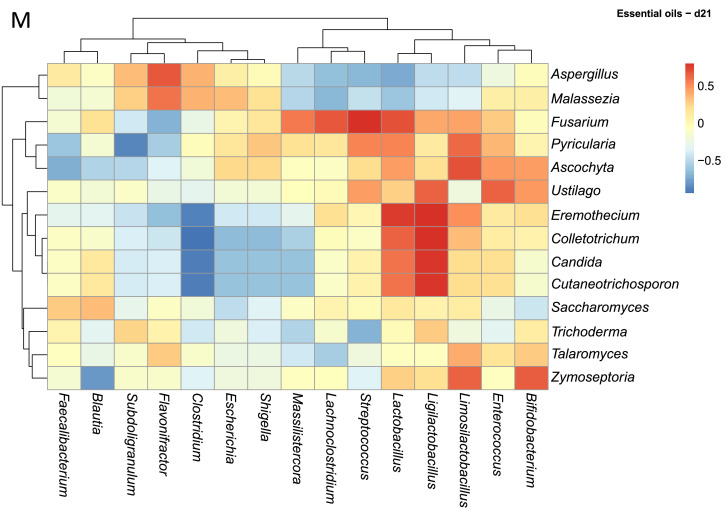
Spearman correlation analysis between the abundances of excreta microbiota and excreta mycobiota. Heatmaps (A-M) visually represent the ecological correlation between fungal and bacterial taxa under different treatments (Basal Diet, Probiotic, Antibiotic, and Essential oils) and developmental stages (d1, d10, d21). Genera on the axes (y axis fungal genera and x axis bacterial genera) and color gradients representing correlation strength and direction. The intensity of the colors represents the degree of correlation. Red represents a significant positive correlation (*P* < 0.05), blue represents significantly negative correlation (*P* < 0.05), and white shows that the correlation was not significant (*P* > 0.05).

Across this dataset (Fig. 6A), fungal mycobiota displayed a spectrum of correlations with specific bacterial taxa, ranging from positive, neutral or negative (Fig. 6A). For example, *Candida* was strongly positively correlated with *Streptococcus* and *Escherichia/Shigella*. Conversely, *Candida* displayed negative correlations with several health-associated bacterial genera, including *Bifidobacterium, Ligilactobacillus, Faecalibacterium, Subdoligranulum, Clostridium, Flavonifractor, Blautia*, and *Lachnoclostridium*. Interestingly, *Pyricularia* exhibited strong positive correlations with the same bacterial genera negatively correlated with *Candida*, suggesting possible competitive or complementary dynamics among fungal members. In contrast, genera such as *Aspergillus* and *Malassezia* appeared to have neutral relationships with bacterial genera like *Faecalibacterium* and *Subdoligranulum*, possibly indicating coexistence.

Temporal variation in fungal–bacterial correlations was evident across treatment groups. In the Basal Diet group at d1 (Fig. 6B), *Talaromyces* showed a strong positive correlation with *Escherichia* and a strong negative correlation with *Clostridium. Saccharomyces* was positively correlated with *Clostridium* but negatively correlated with *Lachnoclostridium, Bifidobacterium*, and *Faecalibacterium*. By d10 (Fig. 6C), these relationships shifted: *Saccharomyces* showed neutral correlations with *Lachnoclostridium, Bifidobacterium*, and *Faecalibacterium*, while exhibiting negative correlations with *Escherichia* and *Shigella*, a pattern that persisted through d21. Additionally, a strong positive correlation emerged between *Saccharomyces, Cutaneotrichosporon, Eremothecium*, and *Bifidobacterium* at d21. Notably, *Fusarium* transitioned from neutral correlations with *Blautia, Massilistercora*, and *Lachnoclostridium* at d10 to strong positive correlations at d21 (Fig. 6D).

In the Probiotic group, d1 (Fig. 6E) was characterized by strong positive correlations between fungal genera such as *Talaromyces, Pyricularia, Trichoderma, Cutaneotrichosporon*, and *Tetrapisispora* and bacterial taxa including *Faecalibacterium, Massilistercora, Subdoligranulum, Flavonifractor*, and *Lachnoclostridium*. In contrast, *Aspergillus* and *Malassezia* showed negative correlations with *Ligilactobacillus, Lactobacillus*, and *Limosilactobacillus*, while *Fusarium* was negatively correlated with *Shigella* and *Faecalibacterium*. As shown in Fig. 6F, at d10 the network expanded, with more widespread positive correlations involving *Talaromyces, Saccharomyces, Ustilago, Cutaneotrichosporon, Ascochyta*, and *Pyricularia* linked to beneficial bacteria such as *Faecalibacterium, Blautia, Subdoligranulum*, and *Massilistercor*a. However, by d21 (Fig. 6G), many of these correlations diminished, suggesting temporal restructuring of fungal–bacterial gut colonization towards a more stable microbiome.

Within the Antibiotic group, d1(Fig. 6H) featured strong positive correlations between the fungal genera *Fusarium, Trichoderma, Malassezia, Zymoseptoria* with the bacterial taxa *Clostridium* and *Lactobacillus*. Meanwhile, *Saccharomyces* and *Tetrapisispora* demonstrated negative (strong/moderate) correlations with many bacteria including *Ligilactobacillus, Limosilactobacillus, Blautia, Bifidobacterium*, and *Faecalibacterium*. By d10 (Fig. 6I), the correlation network broadened; *Ustilago* and *Ascochyta* were positively correlated with *Escherichia, Shigella*, and negatively correlated with *Enterococcus. Malassezia* carried negative correlations with *Shigella* and *Escherichia* from d1 to d10. At d21, the landscape of the two communities became more dynamic. Fungal taxa such as *Trichoderma, Eremothecium*, and *Cutaneotrichosporon* continued to show limited but consistent positive correlations with *Blautia*. Notably, negative correlations involving opportunistic fungi like *Fusarium* and *Malassezia* fluctuated in both strength and frequency (Fig. 6J).

In the Essential oils group, fungal–bacterial populations also exhibited time-dependent shifts at the same time. On d1, *Talaromyces, Cutaneotrichosporon, Pyricularia*, and *Ascochyta* displayed moderate positive correlations with beneficial bacteria including *Faecalibacterium, Ligilactobacillus*, and *Subdoligranulum* (Fig. 6K). Opportunistic fungi such as *Aspergillus, Malassezia*, and *Fusarium* showed negative correlations with *Lactobacillus* and *Bifidobacterium*. By d10 (Fig. 6L), correlations became more diverse: *Trichoderma* was positively associated with *Massilistercora, Lachnoclostridium*, and *Clostridium*, while *Saccharomyces* displayed negative correlations with the same taxa. Interestingly, *Aspergillus* and *Malassezia* showed transient positive correlations with lactic acid bacteria at d10, which reverted to negative by d21. At d21, more fluctuation was observed: positive correlations became concentrated among fungi such as *Fusarium, Ustilago, Pyricularia, Eremothecium, Colletotrichum*, and *Cutaneotrichosporon* with lactic acid producers including *Lactobacillus, Limosilactobacillus*, and *Ligilactobacillus*, while *Aspergillus* and *Malassezia* continued to show weakened but present negative correlations (Fig. 6M). Supplementary table and heatmap with the significant pairs are available on GitHub (https://github.com/aff30/Poultry-Mycobiome-).

### Functional predictions of the excreta mycobiota and microbiota of chickens

**Fig. 7:**
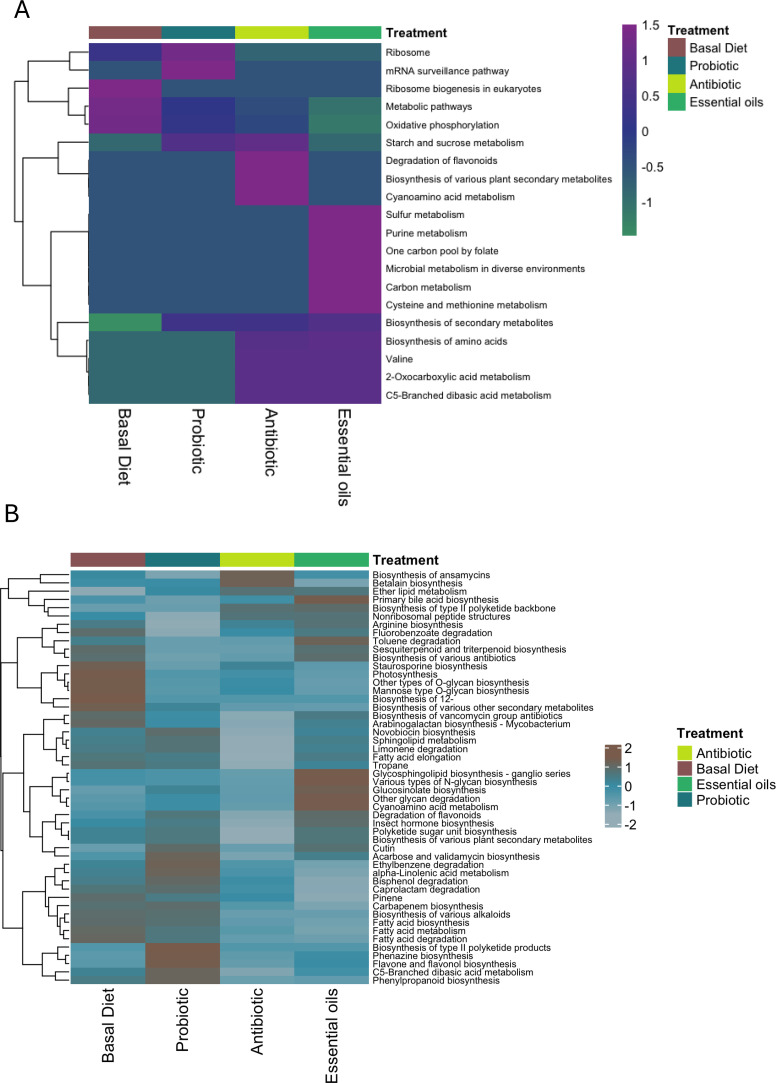
Functional profiles of bacterial and fungal communities in broiler excreta across dietary treatments. (A) Heatmap showing the relative abundance of the top KEGG pathways predicted from the bacterial community, illustrating functional differences among broilers fed a Basal Diet, Probiotic, Antibiotic, or Essential oils treatment. (B) Heatmap showing the relative abundance of the top KEGG pathways predicted from the fungal (mycobiome) community, highlighting functional pathway variations across the same dietary treatments. Color intensity represents z-score standardized pathway abundance values, and hierarchical clustering depicts similarities among treatments and KEGG pathway profiles.

The reads were annotated to total of 259 (fungi) and 3919 (bacteria) KEGG categories. Fig. 7A and B shows the top KEGG pathways with the highest relative abundances. Although no significant differences were seen in the functional profiling of the excreta mycobiome, the clusters of KEGG pathways among Basal Diet and Probiotic groups exhibited higher relative abundance of translation and energy related pathways such as ribosome biogenesis, oxidative phosphorylation, and core metabolic processes, indicating enhanced microbial biosynthetic activity (Fig. 7A). In contrast, the Antibiotic and Essential oils groups showed enrichment of secondary metabolite and amino acid metabolism pathways, reflecting adaptive metabolic responses to antimicrobial or phytogenic feed additives. The bacterial functional profiling did not show significant differences between treatment (Fig. 7B). However, the Antibiotic and Essential oils groups exhibited higher relative abundance of secondary metabolite biosynthesis and xenobiotic degradation pathways, including polyketide, flavonoid, and fatty-acid metabolism, indicating microbial adaptation to antimicrobial or phytogenic compounds. In contrast, the Basal Diet and Probiotic treatments showed greater abundance of amino-acid metabolism pathways, reflecting enhanced metabolic associated functions.

## Discussion

The gut microbiota plays an important role in shaping host physiology, metabolism, immunity, and overall health (Kogut and Zhang, 2022). While the bacterial component of this community has been extensively studied, the fungal component is underexplored, particularly in poultry science (Davies et al., 2022; Robinson et al., 2020, 2022). Emerging research in humans has associated the mycobiome with metabolic disorders and immune modulation, underscoring the need to better understand fungal populations in other species (Huang et al., 2024; Van Syoc et al., 2024). Here, we use shotgun metagenomic sequencing to provide new insights into the temporal dynamics of the excreta mycobiome in broiler chickens and its correlations with excreta bacterial communities under different dietary supplementation strategies. Our findings reveal that fungal community composition is predominantly influenced by bird age rather than feed additive, and that Ascomycota was consistently the dominant phylum across all timepoints and treatments. While alpha diversity remained unaffected by diet, a clear increase in fungal richness and evenness was observed over time, suggesting age-related ecological succession. Beta diversity analyses further supported these temporal trends, showing distinct clustering of fungal communities by age, with notable shifts between days 1, 10, and 21. Moreover, the fungi-bacteria correlation analyses demonstrated dynamic, time-dependent correlation between fungal and bacterial taxa. Together, these findings emphasize the complexity of gut microbial ecosystems and highlight the need for longitudinal, functional, and integrated multi-kingdom approaches to better understand the role of fungi and bacteria in poultry gut health.

The predominance of Ascomycota and Basidiomycota across all treatments and timepoints underscores a conserved fungal core in broiler chickens, in agreement with reports in other species, including humans and pigs (Arfken et al., 2020; Davies et al., 2022; Feng et al., 2023; Robinson et al., 2020, 2022). However, while Robinson et al. (2022) identified Zygomycota as the third most abundant phylum, we observed the presence of Microsporidia*,* a group of spore-forming, obligate intracellular fungi known to infect chicken embryos (Ercan et al., 2020). Such discrepancies in the relative abundance of minor fungal phyla may be explained by differences in sequencing platforms, read depth, bioinformatic pipelines, or reference databases used for fungal taxonomic classification. Additionally, variations in sample type, DNA extraction methods, and environmental or host-associated factors may also contribute to inconsistencies across studies.

Beyond methodological variation, a deeper and often overlooked challenge lies in the inherent instability of fungal taxonomy itself. As highlighted by Money (2013) and Suhr and Hallen-Adams (2015), the field of mycology faces persistent obstacles in achieving accurate and consistent species identification. Money (2013) argues that the traditional nomenclatural system settled Latin terminology and fragmented practices has fostered confusion and redundancy across databases. Suhr and Hallen-Adams (2015) expand this concern to mycobiome studies, noting that next-generation sequencing has amplified these issues by producing large numbers of misidentified or duplicated taxa due to poorly reference libraries. Together, these insights emphasize that inconsistent database management, synonymous naming, and inadequate curation remain major barriers to reliable fungal classification. Addressing these limitations through the development of unified, molecularly informed, and selected taxonomic framework is essential to ensure accurate interpretation of fungal diversity across microbiome studies.

One of the most consistent findings across studies and species is the age-dependent shift in microbiome dynamics (Arfken et al., 2020; Ballou et al., 2016; Oakley and Kogut, 2016). In our previous study, we evaluated the excreta bacterial community of chickens fed feed additives and identified age as a primary driver of bacterial composition and diversity (Fonseca et al., 2024). Expanding on this study, we investigated if excreta-associated fungal composition is also impacted. At d1, fungal communities were dominated by the genera *Candida, Fusarium*, and *Aspergillus*. By days 10 and 21, we observed a marked shift toward more diverse and stable fungal communities, pointing to age-driven mycobiome succession. PCA analysis revealed that *Candida albicans, Aspergillus puulaauensis, Malassezia restricta*, and *Malassezia verticillioides* were the dominant species at d1, whereas *Fusarium pseudograminearum, Aspergillus rugulosus*, and *Malassezia vespertilionis* dominated by day 21. Our study is aligned with Robinson et al. (2020) that also reported intestinal mycobiota succession in chickens. Davies et al. (2022) also showed significant temporal changes in both ileal and cecal fungal communities during the first 14 days post-hatch, with genera such as *Penicillium* and *Meyerozyma* increasing over time. Similarly, Feng et al. (2023) demonstrated that fungal load and diversity increase during early post-hatch development, peaking by day 7 before stabilizing. These patterns suggest that early-life microbial colonization is a critical window for shaping the mycobiome's structure and function.

Environmental variation through the chicken GI tract is another key determinant of mycobiome composition. Robinson et al. (2020) provided a comprehensive biogeographic map of the fungal communities across eight intestinal segments in 28-day-old broilers, revealing that the upper GI tract (crop, ventriculus, duodenum) harbored more diverse fungal communities compared to the lower tract (cecum, colon). Dominant genera included *Scopulariopsis, Trichosporon*, and *Aspergillus*, with a transition in cecal dominance from *Scopulariopsis brevicaulis* to *Trichosporon asahii* between days 14 and 28. While our study did not investigate spatial differences along the GI tract, it offers a novel perspective by characterizing the fungal communities in excreta samples, reflecting the overall output of the GI tract. To better understand the early origin of these fungal taxa, we also analyzed samples from the transport boxes used to deliver chicks from the hatchery. Interestingly, we observed high variability between the three boxes, with *Puccinia, Aspergillus*, and *Malassezia* emerging as the most dominant genera. This variability underscores the stochastic nature of environmental colonization at hatch and highlights the role of immediate surroundings in shaping the initial fungal load.

Here, across all ages and treatments, the most abundant genera were *Candida, Fusarium*, and *Aspergillus*. These genera are frequently reported in poultry mycobiome studies and likely originate from a combination of environmental, dietary, and early-life exposures (Ghaemmaghami et al., 2016; Oliveira et al., 2006; Robinson et al., 2020, 2022). *Candida* specifically is commonly associated with mucosal surfaces and may be acquired immediately post-hatch through contact with eggshells, incubators, or human handlers, as suggested by its high abundance on day 1 in our study. This genus can cause infections in the upper GI tract of chickens which may result in growth impairments and mortality. *Fusarium* species are widespread in cereal grains and are often introduced via feed, especially early in life when gut colonization is still stabilizing (Ghaemmaghami et al., 2016). The presence of *Fusarium* in our samples reflects this possible dietary origin. *Aspergillus* is a ubiquitous environmental genus is commonly found in poultry bedding, dust, and stored grains, and can enter the gut via ingestion or inhalation causing aspergillosis in chickens. Though a possible infectious agent, *Aspergillus* can provide beneficial effects like the production of metabolites such as lovastatin that play a beneficial role in lipid metabolism in chickens (Qureshi et al., 2001).

Like environmental exposures, dietary components, including feed additives and antibiotics, also play a pivotal role gut mycobiome dynamics. However, there were no significant changes in the mycobiota due to treatments interventions in our study. These results reinforce the conclusions drawn by Ward et al. (2019) where feed additives did not impact fungal alpha diversity of turkeys at days 1 or 10. Conversely, Hume et al. (2012) showed that both probiotics and essential oil blends significantly modulated fungal populations in the ileum and cecum, even under *Eimeria* infection. Specifically, probiotics like *Bacillus coagulans* and *Bacillus subtilis*, as well as essential oils, shifted fungal composition away from that of infected controls (Hume et al., 2012). Likewise, Robinson et al. (2020) reported that bacitracin methylene disalicylate administration reduced fungal diversity in the cecum, suggesting antibiotics impact the mycobiome similarly to the bacteriome.

The microbial communities that colonize the GI tract engage in direct or indirect interactions through physical contact, microbial metabolites, and modulation of the host immune system (Pawlowska, 2024). Thus, the fungi-bacteria network might promote ecological and functional dynamics that contribute gut homeostasis maintenance (Coyte et al., 2015; Pawlowska, 2024). Feng et al. (2023) leveraged quantitative PCR and Spearman correlation to show distinct co-occurrence networks between fungal and bacterial taxa, with more positive than negative associations, particularly in the cecum and ileum. Although fungi represented a smaller fraction of gut microbiota than bacteria, their absolute abundance and interactions were non-trivial, contributing to metabolically and potentially influencing host physiology. In our study, we highlight a dynamic and time-sensitive correlation between fungal and bacterial communities in the broiler excreta. Consistent with Iliev and Leonardi (2017) and Robinson et al. (2020), our findings reinforce that mycobiome–microbiome correlations are not static but shift across developmental stages and in response to dietary interventions.

Fungal-bacterial interactions could bring potential implications for gut health, nutrient metabolism, and immune function (Elnagar, 2024). In our study, the genus *Candida* displayed a consistent positive correlation with *Streptococcus* and *Escherichia/Shigella*, both facultative anaerobes frequently associated with early colonization in young chickens (Fonseca et al., 2024; Proszkowiec-Weglarz et al., 2022). Interestingly, Hummel et al. (1973) conducted an in vitro study demonstrating that *Escherichia coli* strains can inhibit fungal colonization, including *Candida albicans*. They proposed that the anti-*Candida* activity of *E. coli* may be mediated not only through immunomodulatory mechanisms but also via the secretion of soluble antifungal factors. Subsequent studies have supported these findings, showing that *E. coli* and its lipopolysaccharide (LPS) can modulate fungal growth and biofilm formation in vitro (Bandara et al., 2009; Cabral et al., 2018). Negative correlations were also observed between *Candida* and health-associated bacteria such as *Bifidobacterium, Faecalibacterium*, and *Ligilactobacillus*. We speculate that this negative correlation signals competitive exclusion, or an indirect antagonism mediated by host immune responses or metabolic byproducts. This co-occurrence may reflect shared metabolic niches, as has been previously observed murine gut models (Fan et al., 2015). For example, *Ligilactobacillus* is a genus of lactic acid bacteria that have the same metabolic niche as *Candida albicans* such that an alteration of *Candida* abundance can change the levels of lactic acid producers like *Ligilactobacillus* (Fan et al., 2015). Moreover, Ricci et al. (2022) demonstrated potential mechanisms driving gut microbiome-mediated colonization resistance against *C. albicans*, identifying particularly inhibitory components such as *Bifidobacterium spp.* and fatty acids as targets.

Unlike *Candida,* the fungal genus *Pyricularia,* showed an opposite correlation profile, positively correlating with many bacterial genera negatively linked to *Candida.* We therefore suggest that there may be potential niche partitioning, the reduction in competition by using gut resources differently, between these fungal genera wherein *Pyricularia* perhaps coexists more harmoniously with beneficial bacteria (Pereira and David Berry, 2016). The neutral correlations observed with *Aspergillus, Fusarium,* and *Malassezia* further support potential commensal behavior within the gut environment. However, to confirm the hypothesis of niche partitioning among these microbes, further analyses using metatranscriptomics or metabolomics would be required to elucidate their active functional profiles and associated metabolic byproducts.

Temporal shifts in fungal composition were evident across all treatment groups. In the Basal Diet group, fungal–bacterial changes evolved from day 1 to day 21. *Saccharomyces* transitioned from negative correlations with *Bifidobacterium* and *Faecalibacterium* to neutral or even positive correlations, suggesting age-dependent modulation of fungal behavior or host immune tolerance (Aruwa and Sabiu, 2024). A similar shift was observed in *Fusarium* wherein a neutral correlation with commensal bacterial genera became a strong positive correlation by day 21, implying potential integration into the stable gut microbiota as chicks aged.

In the Probiotic group, supplementation enhanced positive fungal–bacterial correlations early on. At d1, fungi genera *Talaromyces* and *Trichoderma* were positively linked with key SCFA-producing bacteria, *Subdoligranulum* and *Faecalibacterium*. Some members of the *Trichoderma* genus, are known to secrete cellulolytic enzymes that degrade complex carbohydrates thereby increasing the availability of substrates for SCFA-producing bacteria. This may lead to higher bacterial relative abundance and, consequently, enhance host nutrient digestibility and growth performance (Zhang et al., 2025). Both bacterial and fungal genera are involved in anti-inflammatory and mucosa-supporting roles, suggesting that probiotics may facilitate cooperative cross-kingdom interactions that promote gut health. By d21, however, these correlations diminished, possibly reflecting microbial succession or stabilization of the gut ecosystem in maturing birds.

In contrast, the Antibiotic group presented a more unstable and conflicting dynamic landscape. Early positive correlations between opportunistic fungi (*Fusarium, Trichoderma*) and bacteria such as *Clostridium* may reflect the disruption of bacterial communities and subsequent opportunistic overgrowth of fungi. Persistence of negative correlations between *Saccharomyces* and lactic acid bacteria throughout the study could imply prolonged dysbiosis or competitive exclusion exacerbated by antimicrobial pressure. Essential oils supplementation, however, produced more nuanced effects. Early correlations mirrored those of the Probiotic group, with moderate positive correlations between beneficial fungi and lactic acid bacteria. However, over time, correlations involving *Aspergillus* and *Malassezia* became more transient. These fluctuations might reflect the selective antimicrobial properties of essential oils, which can inhibit certain bacterial or fungal populations while promoting nutrients to others depending on concentration, compound type, and microbial susceptibility (Burt, 2004).

The plasticity of fungal-bacterial changes is further reflected in the functional redundancy observed across the microbiome. Oakley et al. (2014) highlighted that while taxonomic composition can vary widely among individuals, metabolic functions remain conserved, highlighting ecological compensation and resilience. This concept likely extends to fungi, particularly those involved in nutrient fermentation or immune modulation. In our study, the functional predictions derived from KEGG annotations (fungi and bacteria) provide an integrative view of the metabolic potential of the broiler microbiome under different dietary interventions. Consistent with previous poultry metagenomic studies (Jiang et al., 2025; Peña et al., 2025), the dominant functions across all treatments were associated with core metabolic processes, including amino acid, carbohydrate, and energy metabolism, reflecting the fundamental roles of gut microbes in nutrient utilization and host energy balance. The enrichment of ribosome biogenesis**,** oxidative phosphorylation, and carbon metabolism pathways in the Basal Diet and Probiotic groups suggests enhanced microbial growth and biosynthetic activity, likely linked to improved nutrient assimilation and gut homeostasis. In contrast, the Antibiotic and Essential oils treatments showed higher relative abundance of pathways related to secondary metabolite biosynthesis and xenobiotic degradation, such as polyketide, flavonoid, and fatty acid metabolism, indicative of adaptive microbial responses to antimicrobial or phytogenic stressors (Jian et al., 2025). Overall, functional profiling underscores the metabolic resilience of the excreta microbiome and supports the idea that dietary additives influence microbial functionality more subtly than composition, shaping energy production which is critical for maintaining gut health in broiler chickens.

Future research is needed as functional studies specific to the chicken mycobiome and microbiota remain scarce. An intriguing finding by Davies et al. (2022) was that post-hatch feed delay, a common commercial practice, had only transient effects on the mycobiome composition. While differences were noted at day 1 and 2, fungal profiles converged by day 3, suggesting resilience or rapid compensation of fungal communities in the face of short-term nutritional deprivation. This raises questions about the relative sensitivity of fungal and bacterial communities to environmental perturbations. Overall, our data suggest that fungal–bacterial correlations are highly influenced by both age and diet, supporting the concept of a co-evolving gut microbiome that undergoes ecological succession. The presence of strong positive correlations between health-promoting fungi and SCFA-producing bacteria in the Probiotic and Essential oil groups at early time points underscores the potential for microbiome modulators to shape gut microbial networks in beneficial ways. Conversely, the more disorganized correlation patterns under antibiotic pressure highlight the risks of broad-spectrum antimicrobials in destabilizing microbial ecosystems.

### Conclusions

In summary, our study provides evidence that excreta fungal communities in broiler chickens are primarily shaped by age and metabolic functional characteristics. In this study, while the administration of a Probiotic and Essential oils may not dramatically change fungal diversity metrics, they appear to fine-tune microbial correlations. These findings underscore the importance of considering multi-kingdom microbial dynamics when designing strategies to improve poultry health and performance. Future studies should integrate functional assays, such as metabolomics and host immune profiling, to better understand the ecological and physiological implications of these microbial shifts.

## Availability of data and materials

Sequencing data are available at PRJNA928060. All tools used in the analysis are publicly available as described in Methods and References. Performance and microbiome analysis codes, and complementary tables are available at GitHub (https://github.com/aff30/Poultry-Mycobiome-)

## CRediT authorship contribution statement

**Ana Fonseca:** Writing – review & editing, Writing – original draft, Visualization, Validation, Software, Project administration, Methodology, Investigation, Formal analysis, Data curation, Conceptualization. **Sophia Kenney:** Writing – review & editing. **John Boney:** Writing – review & editing. **Erika Ganda:** Writing – review & editing, Supervision, Investigation.

## Disclosures

The authors declare that they have no known competing financial interests or personal relationships that could have appeared to influence the work reported in this paper.
